# Exploring the Conserved Role of MANF in the Unfolded Protein Response in *Drosophila melanogaster*

**DOI:** 10.1371/journal.pone.0151550

**Published:** 2016-03-14

**Authors:** Riitta Lindström, Päivi Lindholm, Jukka Kallijärvi, Mari Palgi, Mart Saarma, Tapio I. Heino

**Affiliations:** 1 Department of Biosciences, University of Helsinki, Helsinki, Finland; 2 Institute of Biotechnology, University of Helsinki, Helsinki, Finland; 3 Folkhälsan Research Center, University of Helsinki, Helsinki, Finland; 4 Department of Gene Technology, Tallinn University of Technology, Tallinn, Estonia; University of Massachusetts Medical School, UNITED STATES

## Abstract

Disturbances in the homeostasis of endoplasmic reticulum (ER) referred to as ER stress is involved in a variety of human diseases. ER stress activates unfolded protein response (UPR), a cellular mechanism the purpose of which is to restore ER homeostasis. Previous studies show that Mesencephalic Astrocyte-derived Neurotrophic Factor (MANF) is an important novel component in the regulation of UPR. In vertebrates, MANF is upregulated by ER stress and protects cells against ER stress-induced cell death. Biochemical studies have revealed an interaction between mammalian MANF and GRP78, the major ER chaperone promoting protein folding. In this study we discovered that the upregulation of MANF expression in response to drug-induced ER stress is conserved between *Drosophila* and mammals. Additionally, by using a genetic *in vivo* approach we found genetic interactions between *Drosophila Manf* and genes encoding for *Drosophila* homologues of GRP78, PERK and XBP1, the key components of UPR. Our data suggest a role for Manf in the regulation of *Drosophila* UPR.

## Introduction

The accumulation of unfolded or misfolded proteins causes disturbances in endoplasmic reticulum (ER) homeostasis, a phenomenon referred to as ER stress. ER stress in turn activates the unfolded protein response (UPR) (reviewed *e*.*g*. in [1–3]). In order to overcome ER stress, UPR leads to attenuation of protein synthesis, enhancement of degradation of unfolded proteins, and activation of specific signalling cascades. These events aim to reduce the overall protein load in the ER and to enhance the protein folding capacity by selective transcription of chaperones. UPR is activated through three signalling cascades by ER transmembrane sensor proteins PERK (PRKR-like endoplasmic reticulum kinase), IRE1 (inositol requiring enzyme 1), and ATF6 (activating transcription factor 6). All of these three proteins are maintained inactive in normal cellular status by binding to the major ER chaperone GRP78/BiP (Glucose-regulated protein 78/Binding immunoglobulin protein). Upon ER stress, GRP78 is dissociated from the sensor proteins which are subsequently activated. The most ancient of these signalling cascades is mediated by IRE1, the sole branch of UPR identified in *Saccharomyces cerevisiae*. IRE1 has kinase activity and endoribonuclease activity needed for degradation of mRNAs in order to relieve the protein synthesis load. IRE1 is also responsible for the unconventional splicing and thus activation of transcription factor XBP1 (X-box Binding Protein-1), a positive regulator of ER chaperone and other UPR related gene expression. Activated PERK attenuates overall protein synthesis through phosphorylating and thus inhibiting EIF2α (eukaryotic translation initiation factor 2, subunit 1 alpha). However, the decreased activation of EIF2α results in an upregulated translation of specific target mRNAs including ATF4 (activating transcription factor 4) [4,5]. The third signalling pathway is mediated through ATF6, a transcription factor activated by its cleavage and translocation to the nucleus.

In *Drosophila*, both IRE1- and PERK-mediated UPR signalling cascades are conserved. The amino acid sequence of the *Drosophila* homologue of ATF6 is highly similar to its mammalian counterpart, but experimental evidence for its involvement in *Drosophila* UPR is lacking [1,2]. Similar to mammals, the expression of *Drosophila* homologue of GRP78, Hsc3 (Heat shock protein cognate 3), is upregulated upon induced ER stress in Xbp1-dependent manner [6–9] but no biochemical data are available to show its association with ER stress sensor proteins.

The MANF/CDNF family of neurotrophic factors was first characterized based on its trophic function on dopaminergic neurons *in vitro* and *in vivo* [10,11]. When injected into the brain, recombinant mammalian MANF (Mesencephalic Astrocyte-derived Neurotrophic Factor) and CDNF (Cerebral Dopamine Neurotrophic Factor) protect and repair dopaminergic neurons in toxin-induced rodent models of Parkinson’s disease (PD) *in vivo* [11–13]. The sole *Drosophila* homologue, DmManf, is expressed in and secreted from glial cells and supports the dopaminergic system in non-cell-autonomous manner [14]. The role of MANF as an extracellular trophic factor is further supported by the evidence that mammalian MANF is protective against ischemic injury in both neurons and cardiomyocytes [15,16]. However, the biology of MANF is not thoroughly understood. Intriguingly, MANF localizes to the ER and has a protective role against ER stress *in vitro* and *in vivo* [17–21]. Additionally, mammalian MANF binds GRP78 in Ca^2+^-dependent manner *in vitro* and this binding may regulate MANF secretion [16]. MANF can be retained in the ER by its C-terminal signal sequence, RTDL in human and RSEL in *Drosophila* [16,22]. Experimental evidence suggests that mammalian MANF interacts with KDEL-R [KDEL (Lys-Asp-Glu-Leu) endoplasmic reticulum protein retention receptor] and that the C-terminal RTDL sequence of MANF is responsible for this interaction [23]. The relevance of KDEL-R as a mediator of the functions of MANF has not been explored *in vivo*, yet. Recently, MANF was also shown to regulate the expression of ER-resident protein CRELD2 [24].

Both *in vivo* and *in vitro* studies have shown that MANF is upregulated after chemically induced ER stress [17,18,25] and by misfolded mutant proteins accumulating in the ER [17,26]. Mammalian MANF expression is activated upon ER stress by ATF6 and XBP1 through an ER stress response element II found in the promoter region of MANF [17,27]. Based on a knockout mouse model, MANF was found to be essential for the survival of pancreatic β-cells and its loss resulted in severe diabetes due to reduction of beta cell mass and activation of UPR in the pancreatic islets [21]. The protective role against 6-OHDA induced and ischemic neuronal damage has been suggested to rise from the ER-related functions of MANF as these processes have been shown to induce ER stress (reviewed in [28,29]) [17,30].

In *Drosophila*, the loss of *DmManf* is associated with upregulated expression of genes involved in UPR [20]. Additionally, the overexpression of DmManf resulted in downregulation of several UPR-related genes [20]. Here we show that, similar to mammalian MANF, the expression of *DmManf* is induced in response to ER stress *in vitro*. Further, we applied the transgenic approaches for gene silencing *in vivo* to reveal genetic interactions between *DmManf* and genes with known functions in the maintenance of ER homeostasis and in UPR.

## Materials and Methods

### Fly Strains

Fly stocks and crosses were maintained at 25°C. The following fly lines were used in the study: w^−^, UAS-*DmManf*^133^ (line L3), UAS-*DmManf*^135^ (line L5) and *DmManf*^*Δ96*^/TM6 Tb Sb EYFP [14], 69B-GAL4 (Bloomington *Drosophila* Stock Center (BDSC) #1774) [31], *da*-GAL4 (BDSC #5460) [32], MS1096-GAL4 (BDSC #8860) [33], *tub*-GAL4/TM6 Tb Sb EYFP (BDSC #5138) [34], UAS-mCD8-GFP (BDSC #5130) [34], UAS-*Hsc3* (BDSC #5843) [35]. T(2;3)SM6a-TM6B Tb translocation balancer (originating from *pnut*^*XP*^/T(2;3)SM6a-TM6B Tb, BDSC #5687) was used in viability studies (referred as SM6-TM6). UAS-RNAi lines (listed in S1 Table) were obtained from BDSC [36] and Vienna *Drosophila* RNAi Center [37]. Adult flies were imaged with ProgRes SpeedXT camera (Jenoptik). Genes were annotated according to Flybase [38].

### Cell Culture

Schneider 2 (S2) cells were cultured in M3-BPYE medium (Shields and Sang M3, 0.5 g/l KHCO_3_, 1.0 g/l yeast extract, 2.5 g/l bactopeptone and 10% fetal bovine serum, pH 6.6) at 25°C. Cells were treated with DMSO, 1 μM thapsigargin (Molecular Probes), 1 mM DTT (Promega) or 10 μg/ml tunicamycin for 20 hours, collected and total RNA was extracted with NucleoSpin^®^ RNA II (Macherey-Nagel). In-column DNase treatment was performed according to manufacturer’s instructions. Samples were collected from three biological replicates. For agarose gel electrophoresis analysis total RNA was extracted with the TRIZOL reagent (Gibco BRL, Life Technologies).

### Quantitative RT-PCR

Larvae were grown at 25°C on apple juice plates with yeast paste and collected 50–54 h after egg laying (AEL) for 2^nd^ instar larval samples or 20–26 h AEL for early 1^st^ instar larval samples. Five wandering larvae were collected for 3^rd^ instar larval samples and each genotype was collected as three biological replicates. Larvae were snap frozen and stored -80°C until RNA extraction. NucleoSpin^®^ RNA II (Macherey-Nagel) was used in extraction and purification of total RNA. In-column DNase treatment was performed according to manufacturer’s instructions. First strand cDNA was synthesized from total RNA (1 μg) using RevertAid Premium Reverse Transcriptase (Thermo Scientific) and Oligo(dT_18_) primer at 53°C according to manufacturer’s instructions. Expression of *DmManf* mRNA was quantified by LightCycler^®^ 480 Real-Time PCR System with Lightcycler 480 SYBR Green I master mix (Roche). Primer pairs and their PCR efficiencies are presented in S2 Table. PCR efficiency (E) of each primer pair was determined from a relative standard curve. Equation E^-Cp^ in which Cp indicates a crossing point was used to calculate relative concentration of mRNA in each sample. *RpL32* was used for normalization. Each sample was analysed as a duplicate.

### Statistical Analysis

Microsoft^®^ Excel Analysis ToolPak (Microsoft^®^ Office Professional Plus 2010) was used for all statistical analyses. For qPCR analyses, two-tailed Student’s t-test was used. For pupal viability studies, Tb^+^ and Tb^-^ pupae were counted, the number of Tb^+^ pupae was divided by the number of all pupae and normalized to experimentally determined ratio from *tub*-GAL4/TM6 Tb Sb EYFP crossed to wild type and to wild type balanced with SM6-TM6 translocation balancer (S3 Table). For preliminary analyses two vials were counted and statistical analysis was done based on six vials with minimum of 40 pupae.

## Results

### *DmManf* Expression is Upregulated in Response to Drug-Induced ER Stress *in vitro*

**Fig 1:**
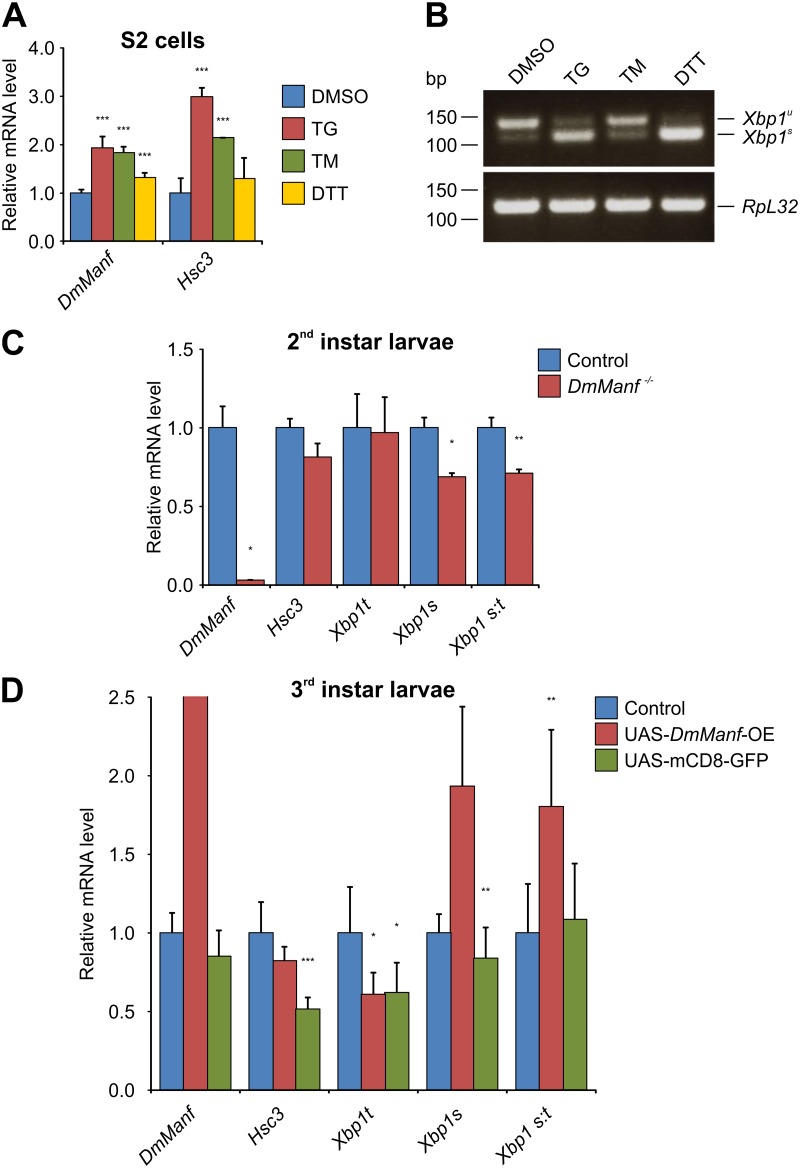
Drug-induced ER stress upregulates *DmManf* expression. A–B) In Schneider 2 (S2) cells, ER stress was induced by thapsigargin (TG), tunicamycin (TM) and dithiothreitol (DTT). DMSO was used as a control treatment. A) The mRNA levels of *DmManf* and *Hsc3* were analysed by qPCR, values were normalised to control treatment (DMSO). B) RT-PCR and agarose gel electrophoresis analysis revealed two transcripts of *Xbp1*, unspliced (*Xbp1*^*u*^) and spliced (*Xbp1*^*s*^). *RpL32* was used as a loading control. C–D) qPCR analysis of *Hsc3* and *Xbp1* expression in *DmManf* mutant (C) and *DmManf* overexpressing (D) larvae. Expression of *Hsc3* was not altered but *Xbp1s* mRNA level was increased in response to overexpression of *DmManf*. The overexpression of *DmManf* resulted in 165-fold increase in *DmManf* mRNA level (±23, P<0.001, not shown). *Xbp1t*, total amount of *Xbp1*; *Xbp1s*, spliced-specific transcript of *Xbp1*; *Xbp1 s*:*t*, proportion of *Xbp1s* out of *Xbp1t*. OE, overexpression. Average ± standard deviation. *, P<0.05; **, P<0.01; ***, P<0.001 versus control, Student’s t-test.

In mammals, MANF is upregulated after chemically induced ER stress [17,18]. To study whether DmManf is involved in *Drosophila* ER stress, we used Schneider 2 cells and induced ER stress by thapsigargin (TG), dithiothreitol (DTT) and tunicamycin (TM). TG depletes Ca^2+^ from the ER by inhibiting Ca^2+^ ATPase [39], DTT reduces the disulphide bridges leading to accumulation of unfolded proteins [40], and TM inhibits N-linked glycosylation [41]. The induction of ER stress was monitored by measuring the mRNA levels of *Drosophila* GRP78 homologue *Hsc3* and *Xbp1* [total (*Xbp1t*) and spliced (*Xbp1s*) forms separately] by qPCR analysis and by evaluating the proportions of unspliced and spliced transcripts of *Xbp1* (*Xbp1*^*u*^ and *Xbp1*^*s*^, respectively) by agarose gel electrophoresis. In agreement with previous studies [6,8], we detected the upregulation of *Hsc3* in response to TG, TM and DTT indicating that ER stress was indeed induced (Fig 1A). Under the control conditions, the *Xbp1u* transcript was prevalent (Fig 1B). TG and DTT treatment induced the splicing of the *Xbp1* transcripts (Fig 1B). The splicing of *Xbp1* was also detected by qPCR analysis (S1A Fig). While total amount of *Xbp1* (*Xbp1t*) was unaltered, the level of *Xbp1s* was increased. Additionally, the ratio of *Xbp1s* to *Xbp1t* (*Xbp1s*:*t*), a commonly used readout of UPR induction, was increased. To study whether the expression of *DmManf* was altered in response to drug-induced ER stress *in vitro* we measured *DmManf* mRNA levels by qPCR analysis. We found that *DmManf* mRNA levels were increased in response to drug-induced ER stress (Fig 1A). These data demonstrated that upregulation of MANF mRNA in response to drug-induced ER stress is conserved between mammals and *Drosophila*.

### Overexpression of *DmManf* Induces Unconventional Splicing of *Xbp1* but does not Alter *Hsc3* Expression

Next, we examined whether altering DmManf expression level induces ER stress *in vivo*. The abolishment of both maternal and zygotic *DmManf* results in lethality at the end of embryogenesis before hatching [14]. Zygotic *DmManf*^*Δ96*^ mutants die during first larval molt due to the persisting maternal contribution of DmManf which promotes the survival of the larvae [14]. In this work, we extracted RNA from larvae at their first larval molt and measured *Hsc3* and *Xbp1* mRNA levels by qPCR. In the zygotic *DmManf*^*Δ96*^ mutant larvae, both *Hsc3* and *Xbp1s* expression was slightly decreased suggesting that UPR was not induced (Fig 1C). The level of *Xbp1t* was not changed.

We also tested if the overexpression of DmManf would affect the mRNA expression of *Hsc3* and *Xbp1*. UAS-*DmManf* construct was driven with ubiquitous *tub*-GAL4 and wandering 3^rd^ instar larvae were collected to gain more long-term *DmManf* overexpression. Overexpression of DmManf did not alter *Hsc3* or *Xbp1t* expression levels, but did increase the amount of *Xbp1s* (Fig 1D). Thus, the *Xbp1s*:*t* ratio was increased as well. This suggests that overexpression of DmManf induces UPR similar to drug-induced ER stress *in vitro* (S1A Fig). However, the mRNA level of *Hsc3* was not altered suggesting that the transcriptional regulation via *Xbp1s* was not activated.

To monitor the extent of overexpression by UAS/GAL4 system, we measured the level of *DmManf* mRNA by qPCR and found massive upregulation of *DmManf* transcript (Fig 1D). Since DmManf enters the secretory pathway, it is possible that any observed effects of DmManf overexpression might be due to increased overall protein synthesis load in the ER and not specifically induced by DmManf. Thus, we used GFP protein with a membrane-directing tag (UAS-mCD8-GFP) as a control for a protein synthesized in the ER. The expression of UAS-mCD8-GFP with *tub*-GAL4 did not increase the expression levels of *Hsc3*, *Xbp1t* or *Xbp1s* (Fig 1D). This suggests that the alterations caused by overexpression of DmManf were not because of the increased overall protein load in the ER but related to DmManf activity.

### *DmManf* Genetically Interacts with Genes Involved in the ER Stress and UPR

**Fig 2:**
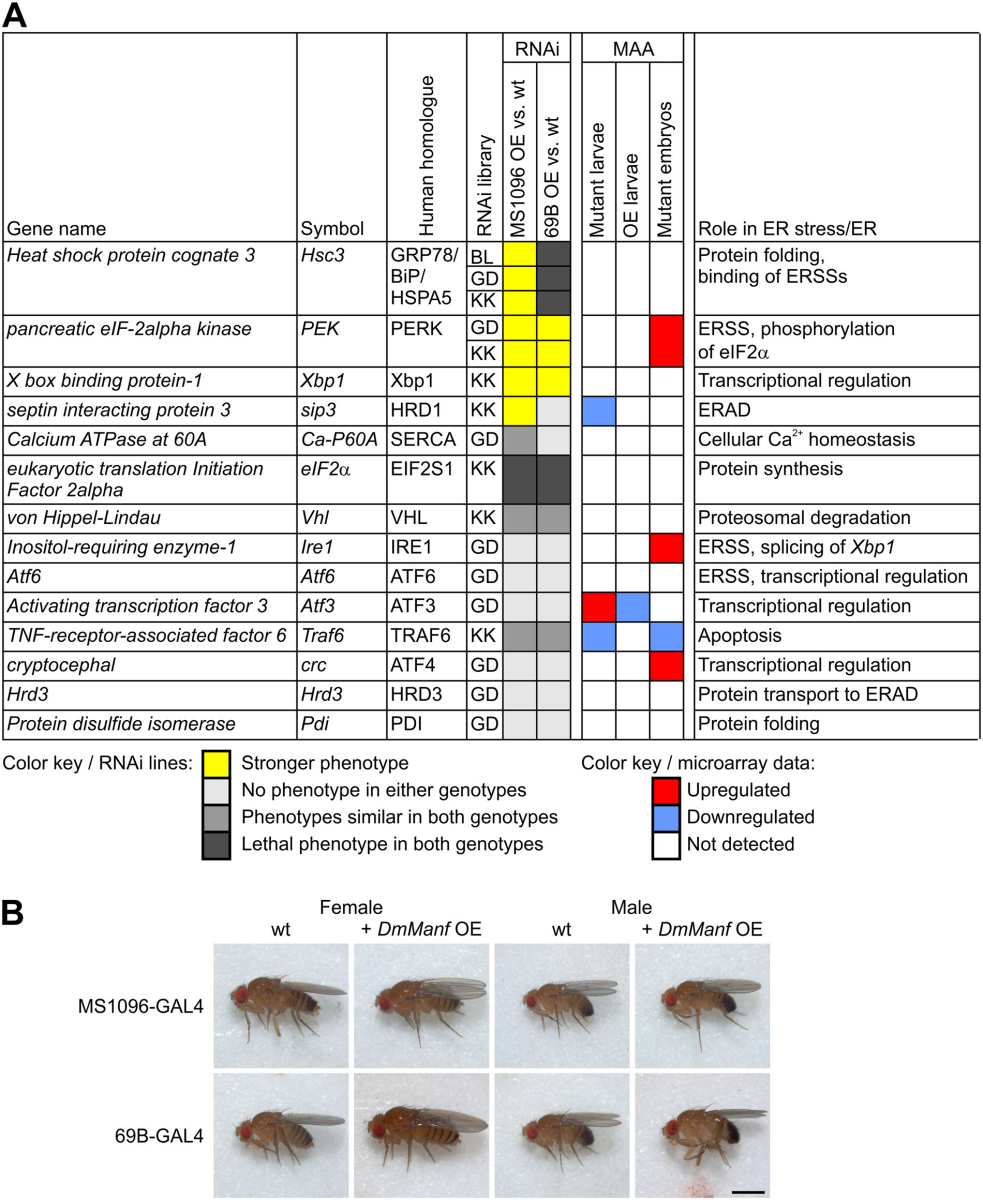
Overview of genetic interactions between *DmManf* and selected ER- and UPR-related genes. A) UAS-RNAi lines were crossed to MS1096-GAL4 and 69B-GAL4 driver lines in wild type and *DmManf*-overexpressing backgrounds. The observed phenotypes of knockdown flies in *DmManf*-overexpressing background (OE vs. wt) were compared to the phenotype of knockdown flies in wild type background. Yellow (stronger phenotype) represents affected phenotypes. Light gray (no phenotype in either background), gray (phenotype similar in both backgrounds) and dark gray (lethal phenotype in both backgrounds) represent cases where the overexpression of *DmManf* did not affect the phenotype caused by knockdown of target gene. As a comparison, results from our previously published microarray analysis (MAA) [20] are presented; red and blue indicate up- and down-regulation of the target gene, respectively. Mutant larvae stands for zygotic *DmManf*^*Δ96*^ mutant larvae, OE larvae for 69B-GAL4>UAS-*DmManf*^L3^ larvae, and mutant embryos for maternal and zygotic *DmManf*^*Δ96mz*^ mutant embryos. B) Overexpression of DmManf by UAS-DmManf^L5^ with either semi-ubiquitous 69B-GAL4 or with wing driver MS1096-GAL4 did not result in any obvious phenotypes in adult flies. In MS1096-GAL4 line, we detected a weak GAL4 expression in CNS as well presenting a probable reason for the lethal phenotypes we observed in our knockdown experiments. However, for screening we only monitored the adult wing phenotype. ER, endoplasmic reticulum; ERAD, ER associated degradation; ERSS, ER stress sensor protein; MAA, microarray analysis; OE, overexpression.

To study further whether *DmManf* interacts with genes involved in the *Drosophila* UPR, we used targeted UAS-RNAi transgenes to inactivate a selected set of genes with known function in *Drosophila* ER and ER stress (Fig 2A). Based on our previous studies, we selected semi-ubiquitous 69B-GAL4 driver [20,31] to knock down target genes for two reasons. First, ectopic DmManf in the 69B-GAL4 expression pattern is sufficient to substitute for the loss of endogenous DmManf protein [14]. Second, by comparing the transgene expression in ubiquitous *da*-GAL4 and semi-ubiquitous 69B-GAL4 pattern we were able to reveal the significance of mutations in the *DmManf* gene for rescuing *DmManf*^*Δ96*^ mutant lethality [22]. In addition to semi-ubiquitous 69B-GAL4 driver, we also wanted to have a more specific expression pattern for the knockdown experiments and used wing driver MS1096-GAL4 [33,42]. We compared the observed phenotypes in wild type and *DmManf*-overexpressing backgrounds to detect whether abundant DmManf expression would affect the knockdown of target genes. The UAS-RNAi lines with distinct phenotypes in these backgrounds were further analysed with ubiquitous *tub*-GAL4 driver to knock down the selected genes with and without *DmManf* overexpression (S3 Table). To verify the specificity of DmManf overexpression on the observed genetic interactions, we used UAS-mCD8-GFP construct as a control for overexpression of a protein processed in the ER. In our previous and current work, we have never detected any obvious phenotypes in flies overexpressing *DmManf* with a variety of GAL4 drivers in wild type background (Fig 2B and S2A Fig, [20,43]). Surprisingly, simultaneous overexpression of DmManf strongly enhanced the phenotypes caused by knockdown of *Hsc3*, *PEK* (pancreatic eIF-2alpha kinase, homologue to human PERK), *Xbp1* and *sip3* (septin interacting protein 3) (Fig 2A, these results are described in detail below).

### *DmManf* Interacts with *Hsc3*, *Drosophila* Homologue of *GRP78*

**Fig 3:**
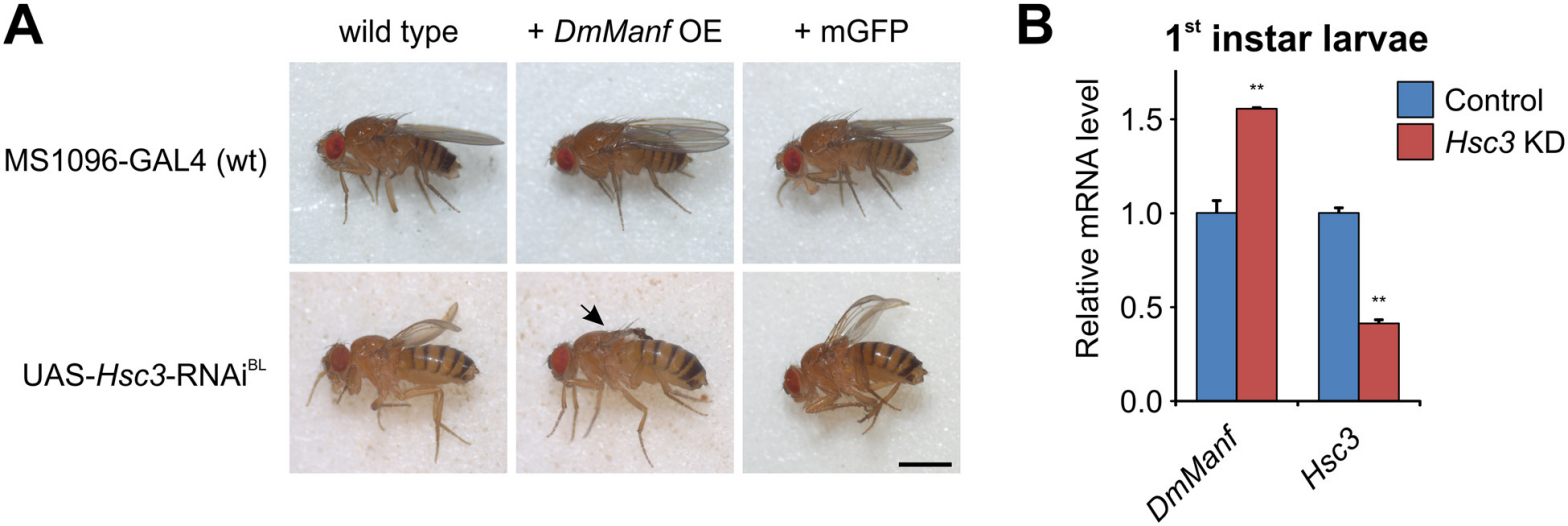
*DmManf* genetically interacts with *Hsc3*, the *Drosophila* homologue of the mammalian ER chaperone GRP78. A) In wild type background, silencing of *Hsc3* by UAS-RNAi construct with the wing driver MS1096-GAL4 resulted in smaller and strongly curled wings. Simultaneous overexpression of *DmManf* (+ *DmManf* OE) enhanced this phenotype and led to a complete disruption of the wings. The simultaneous expression of UAS-mCD8-GFP (+ mGFP) did not alter the phenotype. Scale bar 1 mm. B) Knockdown of *Hsc3* with the 69B-GAL4 driver upregulated *DmManf* mRNA expression in early 1^st^ instar larvae determined by qPCR analysis. Expression level of *Hsc3* was decreased indicating successful silencing of the gene. KD, knockdown; OE, overexpression. Average ± standard deviation. **, P<0.01 versus control, Student’s t-test.

Knockdown of *Hsc3* in the wing with MS1096-GAL4 driver in wild type background resulted in severely malformed wings (Fig 3A). This wing phenotype was further worsened when *DmManf* was simultaneously overexpressed whereas it was not affected by simultaneous expression of UAS-mCD8-GFP (Fig 3A). The knockdown of *Hsc3* with semi-ubiquitous 69B-GAL4 and ubiquitous *tub*-GAL4 drivers was lethal in both wild type and *DmManf*-overexpressing backgrounds (Fig 2A and S3 Table).

To investigate whether silencing of *Hsc3* affected endogenous *DmManf* expression, we knocked down *Hsc3* and analysed *DmManf* mRNA levels by qPCR analysis. The knockdown of *Hsc3* with *tub*-GAL4 resulted in lethality prior to 2^nd^ instar larval stage (S3 Table). Thus, we analysed the *DmManf* mRNA levels at the early 1^st^ larval stage and selected the semi-ubiquitous 69B-GAL4 driver instead of ubiquitous *tub*-GAL4. Although the UAS/GAL4 system is activated [31] and gene expression silenced by UAS-RNAi only from the embryonic stage 9 onwards, we detected a notable decrease in *Hsc3* mRNA levels. The mRNA levels of *DmManf* showed a 1.5-fold increase (Fig 3B) indicating that the knockdown of *Hsc3* resulted in upregulation of *DmManf* expression. These data demonstrated that the genetic interaction between MANF and Hsc3/GRP78 is conserved between *Drosophila* and mammals.

### DmManf and Hsc3 do not Complement each other

Ubiquitous knockdown of *Hsc3* in wild type background was lethal before 50 hours AEL (S3 Table) and this lethality was not rescued by simultaneous overexpression of *DmManf* (S3 Table). To further study the genetic interaction between *DmManf* and *Hsc3*, we used a UAS-*Hsc3* construct to overexpress *Hsc3* [35]. Ubiquitous overexpression of *Hsc3* with *tub*-GAL4 driver in wild type background did not affect overall viability and no obvious phenotypes were detected in the adult flies (S2A–S2C Fig). Furthermore, simultaneous overexpression of *Hsc3* and *DmManf* did not affect viability and showed no obvious phenotype in the emerged adults (S2A–S2C Fig).

The loss of zygotic *DmManf* results in lethality at early larval stage [14]. To examine whether overexpression of *Hsc3* could complement for the lack of DmManf, we crossed UAS-*Hsc3*; *DmManf*^*Δ96*^/SM6-TM6 males to *da*-GAL4 *DmManf*^*Δ96*^ /TM6 females. We could not detect homozygous *DmManf*^*Δ96*^ mutant pupae or adults (number of pupae analysed = 141). Thus, the ubiquitous overexpression of *Hsc3* failed to rescue *DmManf*^*Δ96*^ mutant lethality. Taken together, these data indicate that both Hsc3 and DmManf are necessary for fly viability but unable to complement each other.

### Genetic Interaction between *DmManf* and ER Stress Sensor *PEK*

**Fig 4:**
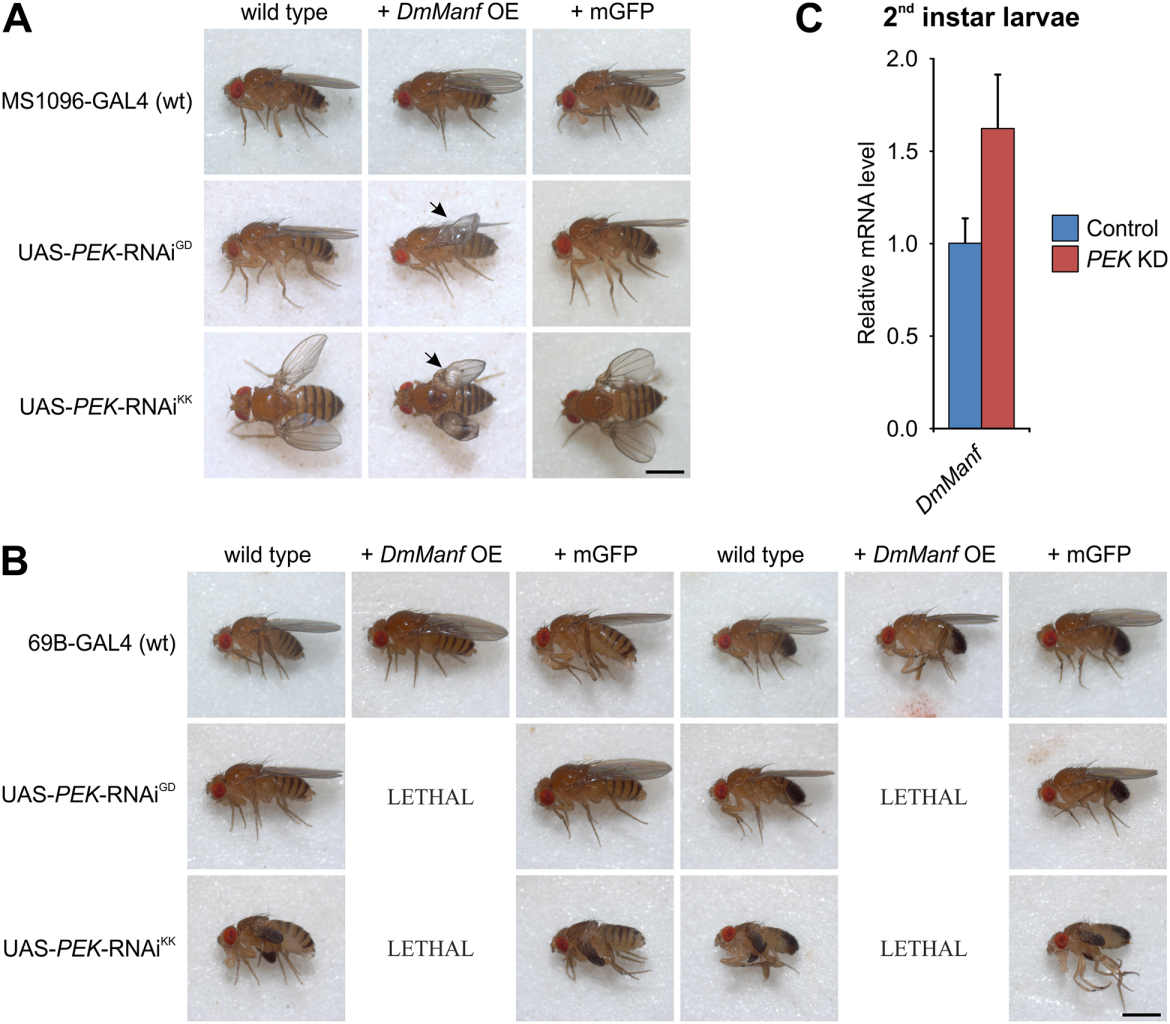
*PEK* genetically interacts with *DmManf*. A–B) The knockdown of *PEK* with the wing driver MS1096-GAL4 (A) and semi-ubiquitous driver 69B-GAL4 (B) resulted in stronger phenotype when *DmManf* was simultaneously overexpressed (+ *DmManf* OE) in comparison to wild type background. UAS-mCD8-GFP (+ mGFP) did not affect *PEK* knockdown. Scale bar 1 mm. C) Quantitative RT-PCR analysis of knockdown of *PEK* with ubiquitous *tub*-GAL4 driver in 50–54 h AEL larvae. Knockdown of *PEK* resulted in increased *DmManf* mRNA levels. KD, knockdown; OE, overexpression. See also S2 Fig for further data. Average ± standard deviation.

Knockdown of *PEK* with MS1096-GAL4 showed more severe wing phenotype together with *DmManf* overexpression in comparison to wild type background (Fig 4A). With semi-ubiquitous 69B-GAL4 driver, the knockdown of *PEK* was viable in wild type background (Fig 4B). However, together with *DmManf* overexpression the knockdown of *PEK* with 69B-GAL4 was lethal at pupal stage (Fig 4B). In wild type background the ubiquitous knockdown of *PEK* was viable (S2D and S2E Fig, S3 Table). Interestingly, simultaneous overexpression of *DmManf* worsened the ubiquitous knockdown of *PEK* to lethality at larval stage (S2D Fig, S3 Table). Simultaneous expression of UAS-mCD8-GFP did not affect the *PEK* knockdown either by MS1096-GAL4, 69B-GAL4 or *tub*-GAL4 drivers (Fig 4A and 4B, S3 Table) indicating that the observed changes caused by overexpression of DmManf were due to increased DmManf activity.

To verify the knockdown of *PEK* expression by UAS-*PEK*-RNAi construct, we measured the *PEK* transcript levels by qPCR analysis in ubiquitous *PEK* knockdown larvae 50–54 hours AEL. Indeed, the expression level was significantly decreased (S1B Fig). To further elaborate the genetic interaction of *DmManf* with *PEK*, we investigated the effect of ubiquitous knockdown of *PEK* on *DmManf* expression by analysing *DmManf* mRNA levels in *PEK* knockdown larvae. Interestingly, ubiquitous knockdown of *PEK* increased the mRNA levels of *DmManf* (Fig 4C). This qPCR analysis together with the *in vivo* phenotypic data indicates genetic interaction between *DmManf* and *PEK*, the functionally conserved *Drosophila* homologue of mammalian UPR transducer PERK.

### Overexpression of *DmManf* Alters *Xbp1* Knockdown Phenotype

**Fig 5:**
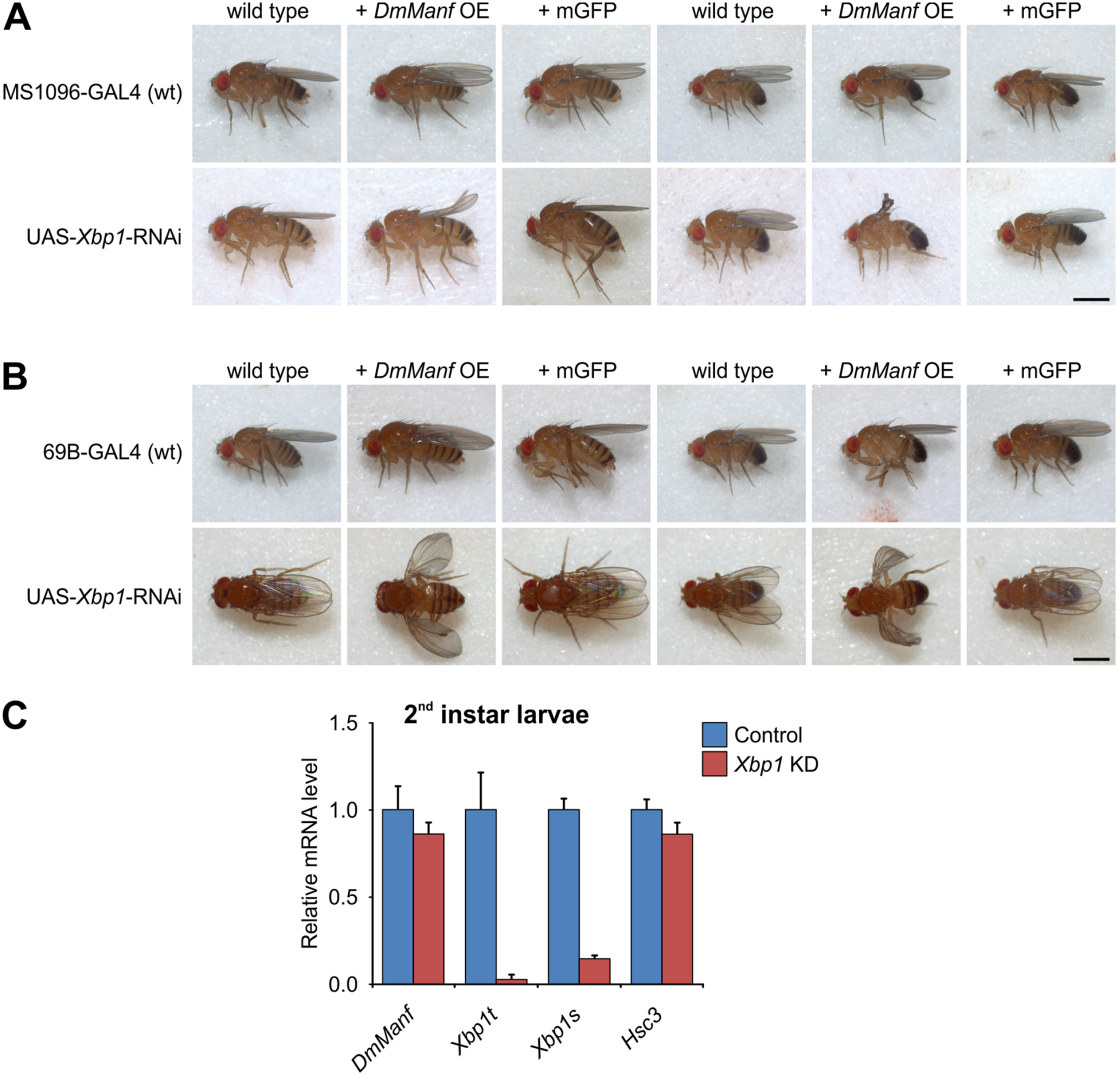
*Xbp1* is a genetic interactor of *DmManf*. A–B) The knockdown of *Xbp1* with wing driver MS1096-GAL4 (A) and semi-ubiquitous 69B-GAL4 driver (B) resulted in stronger phenotype when *DmManf* was simultaneously overexpressed (+ *DmManf* OE) in comparison to wild type background. Simultaneous expression of UAS-mCD8-GFP (+ mGFP) did not affect the *Xbp1* knockdown phenotypes. Scale bar 1 mm. C) Quantitative RT-PCR analysis of knockdown of *Xbp1* with ubiquitous *tub*-GAL4 driver in 50–54 h AEL larvae. Knockdown of *Xbp1* showed minimal decrease in *DmManf* mRNA level and small decrease in *Hsc3* levels. The amounts of both total and spliced *Xbp1* transcripts were clearly decreased indicating a successful knockdown of *Xbp1* gene. KD, knockdown; OE, overexpression. See also S2 Fig for further data. Average ± standard deviation.

In wild type background, the knockdown of *Xbp1* with either wing driver MS1096-GAL4 or semi-ubiquitous 69B-GAL4 showed barely notable phenotype in adult wings (Fig 5A and 5B). When *Xbp1* was knocked down with these drivers together with *DmManf* overexpression, the adults showed clearly stronger wing phenotypes (Fig 5A and 5B). In wild type background the ubiquitous knockdown of *Xbp1* with *tub*-GAL4 was partially lethal at larval stage (S2D and S2E Fig, S3 Table). In *DmManf*-overexpressing background, ubiquitous knockdown of *Xbp1* resulted in complete larval lethality (S2D Fig, S3 Table). Simultaneous expression of UAS-mCD8-GFP did not alter *Xbp1* knockdown with any of the GAL4 drivers used (Fig 5A and 5B, S3 Table).

Again, we quantified by qPCR the expression level of *DmManf*, *Hsc3* and *Xbp1* mRNAs in ubiquitous *Xbp1*-knockdown larvae. Both total amount and spliced form of *Xbp1* mRNA showed severely reduced expression level indicating a successful knockdown by UAS-*Xbp1*-RNAi transgene (Fig 5C). The ubiquitous knockdown of *Xbp1* with *tub*-GAL4 only slightly reduced the *DmManf* and *Hsc3* mRNA levels (Fig 5C). However, overexpression of *DmManf* in wild type background resulted in increased level of *Xbp1s* transcript (Fig 1D). Thus, the data presented in this study strongly indicated that *DmManf* and *Xbp1* interact with each other.

Upon UPR, the splicing of Xbp1 is carried out by Ire1, one of the ER stress sensor proteins. In our study, we also examined the genetic interaction between *DmManf* and *Ire1*. The knockdown of *Ire1* with MS1096-GAL4, 69B-GAL4 and *tub*-GAL4 did not show any obvious phenotype in the adult flies either in wild type and *DmManf*-overexpressing backgrounds (Fig 2A). The *Ire1* mutant larvae die prior 72 h AEL [44]. Most likely, the knockdown by UAS-*Ire1*-RNAi construct was insufficient to reduce the *Ire1* expression level enough to detect any genetic interactions in this study.

### *DmManf* Interacts with *sip3*, a Gene Encoding for a Component of ER-Associated Degradation (ERAD)

The simultaneous overexpression of *DmManf* also affected the knockdown of *sip3* (septin interacting protein 3) (Fig 2A). *sip3* encodes for the *Drosophila* homologue of human synoviolin/HRD1, an ER resident E3 ubiquitin ligase with specific function in ERAD. In wild type background, the knockdown of *sip3* with wing driver MS1096-GAL4 showed subtly uneven wing phenotype (S2F Fig). The simultaneous overexpression of *DmManf* enhanced this phenotype to mildly wrinkled wings (S2F Fig). Co-expression of UAS-mCD8-GFP did not affect the *sip3* knockdown phenotype (S2F Fig). The ubiquitous knockdown of *sip3* with *tub*-GAL4 resulted in lethality prior to pupal stage in both wild type and *DmManf*-overexpressing backgrounds (S3 Table). In our previous study we found that *sip3* was downregulated in *DmManf*^*Δ96*^ mutant larvae. Taken together, these data demonstrated a genetic interaction between *DmManf* and *sip3*.

### *DmManf* does not Genetically Interact with the ER Stress Sensor Atf6

Previous studies on mammalian systems have suggested an interaction between MANF and ATF6, one of the three ER stress sensor proteins [17,27]. In the current study, we found a genetic interaction between *DmManf* and *PEK*, a gene encoding for another ER stress sensor protein. We did not detect any phenotype in the *Atf6* knockdown flies by either MS1096-GAL4, 69B-GAL4 or *tub*-GAL4 drivers (Fig 2A). Simultaneous overexpression of DmManf did not affect *Atf6* knockdown by MS1096-GAL4 or 69B-GAL4 drivers (Fig 2A). We also measured the *Atf6* mRNA levels by qPCR in *DmManf* mutant (S1C Fig) and overexpressing (S1D Fig) larvae and could not detect alterations in the *Atf6* expression. Further, we analysed the *DmManf* mRNA level by qPCR analysis in ubiquitous *Atf6* knockdown larvae. The *DmManf* expression level was not altered while *Atf6* expression level was decreased (S1C Fig). Taken together, our data suggest that there is no genetic interaction between *DmManf* and *Atf6* in this experimental setup.

## Discussion

**Fig 6:**
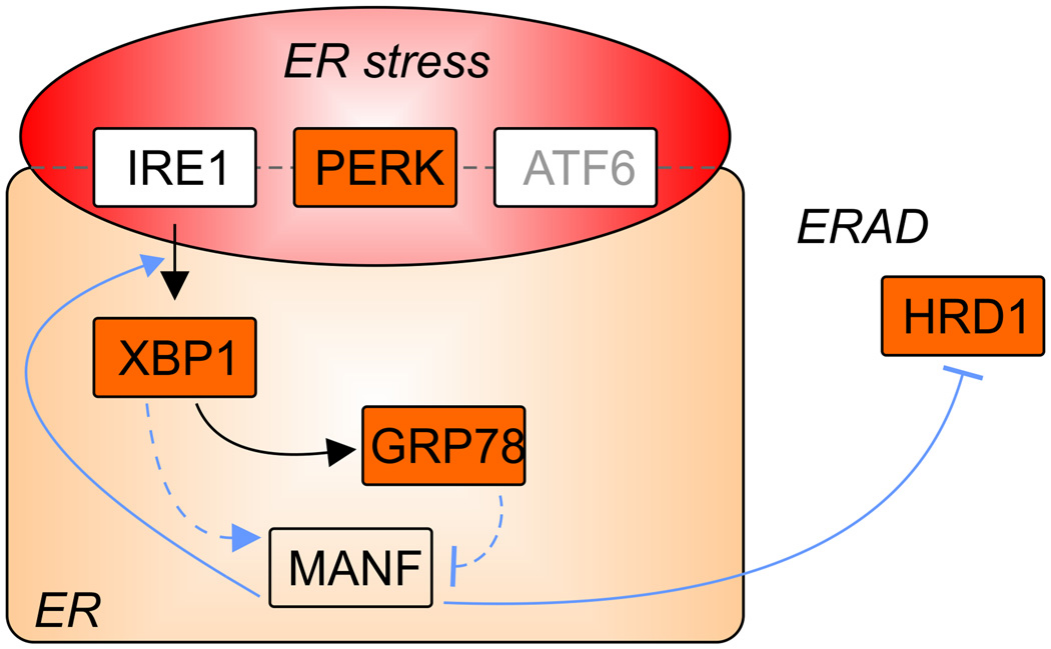
A simplified presentation of UPR and genetic interactions (coloured in orange) discovered for *Drosophila Manf*. ER stress sensor proteins IRE1, PERK and ATF6 reside on ER membrane. The role of ATF6 in *Drosophila* UPR is uncharacterised (in gray). Upon ER stress, transcription factor XBP1 is spliced by IRE1 and activated. XBP1 directs expression of chaperones, including GRP78. In cultured mammalian cells, XBP1 is also suggested to regulate MANF expression. HRD1, an ER resident E3 ubiquitin ligase, functions in ERAD, a process in which terminally misfolded proteins are degraded in the cytosol. In this study, we found that DmManf genetically interacts with Hsc3/GRP78, Xbp1, Pek/PERK and sip3/HRD1 but the functional role of DmManf in *Drosophila* UPR remains to be solved in future. In blue lines are presented the regulatory genetic interactions our data suggest. Dashed lines indicate speculative interactions.

Increasing evidence indicates that ER stress and UPR play a major role in variety of human diseases including diabetes mellitus and neurodegenerative disorders (reviewed e.g. in [3,45]). MANF is a secreted protein [14,30], but also localizes to the ER and has a role in mammalian UPR [17–20]. In this study, we examined the role of DmManf in UPR in the *Drosophila* model. We show that the upregulation of MANF mRNA expression by ER stress-inducing agents is conserved in *Drosophila* S2 cells. Additionally, we found genetic interaction between *DmManf* and genes known to function in the ER and UPR. A schematic presentation of the interactions discovered is presented in Fig 6.

One of the interacting partners was *Hsc3*, the *Drosophila* homologue of mammalian chaperone GRP78. The silencing of *Hsc3* in the wing resulted in an abnormal wing phenotype in wild type background. This wing phenotype was stronger in *DmManf*-overexpressing background. In cultured mammalian cells MANF has been shown to bind GRP78 in Ca^2+^-dependent manner and the loss of interaction between mammalian MANF and GRP78 was associated with increased secretion of MANF [16]. In line, the knockdown of *Hsc3* could lead to increased secretion of DmManf and lead to deprivation of intracellular DmManf. In our previous study, we noticed that the deletion of ER retention signal RSEL increased the secretion of DmManf in S2 cells and decreased its functionality in rescue experiments *in vivo* [22]. Based on the physical interaction found between mammalian MANF and GRP78, the simultaneous overexpression of *DmManf* and knockdown of *Hsc3* could also result in the abundant DmManf binding the residual Hsc3 and preventing other important cellular functions of Hsc3. Alternatively, the loss of *Hsc3* could lead to decreased protein folding capacity in the ER and activation of UPR. The vast amount of DmManf protein could exhaust this already disturbed cellular state.

In previous studies, mammalian MANF has been suggested to have chaperone-like functions, *e*.*g*. by binding unfolded proteins *in vitro* [46] but the putative chaperone activity remains unconfirmed [17,46]. The major ER chaperone Hsc3 and DmManf clearly have distinct roles as either the overexpression or the loss of one could not complement for the loss of the other. However, our study indicates that the interaction between MANF and GRP78 [16] is conserved. In future, the functional significance of this intriguing interaction deserves to be addressed in detail.

We also found that *DmManf* genetically interacted with *PEK*/*PERK*, an ER stress sensor protein. Similar to the silencing of *Hsc3*, we found that simultaneous overexpression of *DmManf* worsened the phenotypes observed in *PEK* knockdown flies. Previous studies have indicated functional conservation of PERK in *Drosophila* and mammals (reviewed in [1,2]). The *Drosophila* homologue to ATF4, the downstream target of activated PERK and selectively upregulated by UPR, showed no genetic interaction with *DmManf* in our study (Fig 2A). We have previously shown that the abolishment of both zygotic and maternal *DmManf* resulted in increased phosphorylation of eIF2α, another molecular marker used for detecting ER stress [20]. In this study, we abolished only the zygotic *DmManf* while maternal *DmManf* was still present. The loss of zygotic *DmManf* alone did not induce UPR when evaluated by other readouts, *i*.*e*. increased *Hsc3* mRNA level and splicing of *Xbp1*. Although the zygotic *DmManf*^*Δ96*^ mutant larvae show only low amount of persisting maternal *DmManf* mRNA and protein (Fig 1C and [14]), it could be sufficient to prevent the induction of UPR.

Additionally, we discovered a genetic interaction between *DmManf* and *Xbp1*, a transcription factor mainly responsible for the regulation of UPR-induced genes. Upon UPR, the mRNA of *Xbp1* is spliced by IRE1 and translated into a transcriptional activator of chaperone expression in response to the increased protein folding demand [47,48] (reviewed *e*.*g*. in [49]). According to previous studies, the spliced form of Xbp1 could mediate the UPR-induced upregulation of MANF in mammals [27,50,51]. MANF has been suggested to have protective role against ER stress [15,16,18,21,30,46]. During normal development, ER stress is detected in the secretory cells and the silencing of *Xbp1* disturbs this developmental ER stress [44,52–54]. Both mammalian and *Drosophila* MANF has been shown to have especially high expression levels in secretory tissues [14,17,20,30]. We found that overexpression of *DmManf* increased *Xbp1s* mRNA level but the knockdown of *Xbp1* did not affect *DmManf* expression levels. Also, the mRNA levels of *Hsc3* were not upregulated in *Xbp1* knockdown larvae. This could indicate the lack of transcriptional activation of *DmManf* and *Hsc3* expression by Xbp1s in *Xbp1*-knockdown larvae. Therefore, knockdown of *Xbp1* could compromise the regulation of *DmManf* expression in the developmental ER stress and deteriorate its function in the secretory cells.

ERAD is a cellular process aiming to clear out the unfolded and misfolded proteins from the ER (reviewed e.g. in [55]). According to our previous transcriptome analysis, *sip3* was downregulated in *DmManf*^*Δ96*^ mutant larvae [20]. In this study, we also found a genetic interaction between *DmManf* and *sip3*. *Sip3* encodes for a homologue to mammalian ER resident E3 ubiquitin ligase synoviolin/HRD1 with specific function in ERAD. Mammalian MANF is upregulated by ERSE-II (ER stress response element II) found in its promoter region [17]. Interestingly, ERSE-II is also found in ERAD-related components HERPUD1 (homocysteine-inducible, ER stress-inducible, ubiquitin-like domain member 1, also known as HERP) [56] and VIMP (VCP-interacting membrane protein, also known as selenoprotein S) [57]. ERSE-II has been hypothesized to regulate the protein quality control and degradation of misfolded proteins during ER stress suggesting that MANF could also have a role in these functions [17].

Surprisingly, we discovered that the overexpression of *DmManf* led to enhanced phenotypes in flies of which a UPR-related gene was knocked down. Thus far, overexpression of *DmManf* with any GAL4 driver we have tested has never resulted in a detectable phenotype or altered viability (Fig 2B, S2A Fig, [20,43]). According to our previous microarray analysis, *DmManf* overexpression led to downregulation of UPR-related genes [20]. This suggests that the overexpression of DmManf would disturb UPR signalling. Hypothetically, in wild type background cells would be able to deal with the increased DmManf expression and the subsequent downregulation of UPR-related genes whereas the additional knockdown of an important component of UPR, e.g. *Hsc3*, *PEK* or *Xbp1*, could compromise the cell homeostasis.

An alternative explanation for our observations in interaction studies between UPR genes and *DmManf* would be that DmManf is actually a substrate for UPR. Then, the abundant expression of DmManf by UAS/GAL4 would rather model the effects of increased overall protein synthesis in ER than indicate specific ER-related functions for DmManf. DmManf enters the secretory pathway [14] and its ectopic expression may cause stress to the protein folding machinery in the ER. Although the *Xbp1s* mRNA level was increased, the expression of *Hsc3* was not altered indicating that overexpression of DmManf induces mild UPR. However, we did not see similar effects with overexpression of membrane-directed GFP suggesting that the observed phenomena were specific for DmManf.

In our previous microarray study, we found that the total loss of *DmManf* is associated with upregulated expression of genes involved in UPR [20]. However, in the current study we found that the mRNA levels of *Hsc3* and *Xbp1* were mildly decreased in *DmManf* mutant larvae. In the previous study, transcriptome analysis was done from the embryonic *DmManf* mutants lacking both maternal and zygotic *DmManf*. In the current study, we collected RNA from zygotic *DmManf* mutants with the persisting maternal *DmManf* mRNA and protein [14]. The maternal *DmManf* is apparently sufficient to prevent induction of UPR and upregulation of UPR related genes.

This work provides evidence for the contribution of DmManf in *Drosophila* UPR. Further biochemical studies on the interaction between *DmManf* and UPR genes in *Drosophila* are needed to elucidate the details of this process.

## Supporting Information

S1 Fig Additional qPCR analyses. A) In Schneider 2 (S2) cells treated with ER stress-inducing drugs thapsigargin (TG), tunicamycin (TM) and dithiothreitol (DTT) the mRNA level of *Xbp1s* is increased while *Xbp1t* remains unaltered resulting in increase of *Xbp1 s*:*t* ratio. *Xbp1t*, total amount of *Xbp1*; *Xbp1s*, spliced-specific transcript of *Xbp1*; *Xbp1 s*:*t*, proportion of *Xbp1s* out of *Xbp1t*. B) In ubiquitous *PEK* knockdown larvae, mRNA level of *PEK* was decreased. C) The *Atf6* mRNA level was not altered in zygotic *DmManf* mutants. Ubiquitous knockdown of *Atf6* showed decreased expression level of *Atf6* but did not alter *DmManf* mRNA expression. D) In 3^rd^ instar wandering larvae, ubiquitous *DmManf* overexpression did not affect *Atf6* expression. KD, knockdown. Average ± standard deviation. *, P<0.05; **, P<0.01; ***, P<0.001 versus control, Student’s t-test.
(TIF)

S2 Fig Phenotypic analyses of *DmManf* genetic interactors. A–C) Ubiquitous overexpression of *Hsc3* does not affect fly viability. A) Overexpression of *DmManf* or *Hsc3* with *tub*-GAL4 showed no phenotype in adult flies. B–C) Viability of *Hsc3* overexpression pupae (B) or adults (C) was not affected by overexpression of *DmManf*. D–E) Ubiquitous knockdown of *PEK* and *Xbp1* with *tub*-GAL4 was viable (*PEK*) and only partially lethal (*Xbp1*) at pupal stage (D). Knockdown of *PEK* was also partially viable at adult stage (E). With *DmManf* overexpression, the ubiquitous knockdown of both *PEK* and *Xbp1* was completely lethal at larval stage. F) Knockdown of *sip3* with wing driver MS1096-GAL4 results in wrinkled wing phenotype in *DmManf*-overexpressing background. Scale bar 1 mm (in A and F). Amount of pupae analysed in B–C and D–E are presented in S3 Table. Proportion of Tb+ pupae was normalized to experimentally determined proportion of Tb+ pupae (see S3 Table, wild type and wild type/SM6-TM6). OE, overexpression.
(TIF)

S1 Table List of UAS-RNAi lines used in the study. Symbols used: Tf ID, transformant line identification; Collection, RNAi library where GD = Vienna *Drosophila* RNAi Center (VDRC) GD library, KK = VDRC KK library, BL = TRiP-3 collection available in Bloomington *Drosophila* Stock Center. According to the VDRC datasheet, the UAS-*Xbp1*-RNAi construct in transformant line 109312 targets both unspliced and spliced *Xbp1* transcripts.
(DOCX)

S2 Table List of primer pairs used in the qPCR analysis and their PCR efficiencies (E). *Hsc3* and *Pek* primers were designed with FlyPrimerBank (http://www.flyrnai.org/FlyPrimerBank) [58]. *DmManf* and *RpL32* were adopted from [20], *Xbp1t* from [59], *Xbp1s* from [60] and *Atf6* from [61].
(DOCX)

S3 Table Results from ubiquitous knockdown studies of UAS-RNAi lines. *tub*-GAL4/TM6 Tb Sb EYFP females were crossed to UAS-*x*-RNAi (wild type background), UAS-*x*-RNAi; UAS-*DmManf*-OE/SM6-TM6 (+ UAS-*DmManf*-OE) or UAS-*x*-RNAi; UAS-mCD8-GFP/SM6-TM6 (+ UAS-mCD8-GFP) males. Since UAS-*Hsc3*-RNAi^BL^ construct was inserted in 3^rd^ chromosome and the insertion was lethal, UAS-*Hsc3*-RNAi^BL^/SM6-TM6 and UAS-*DmManf*-OE; UAS-*Hsc3*-RNAi^BL^/SM6-TM6 males were used. Columns: Tb+ and Tb-, amounts of Tb+ and Tb- pupae in crosses; Pupae, normalized proportion of Tb+ of all pupae, wild type or wild type/SM6-TM6 were used to normalize proportions; Adults, proportion of emerged adults out of Tb+ pupae. OE, overexpression; ND, not determined. n of analysed vials = 6 (except ^1^, n = 2 vials).
(DOCX)
