# Supplementary figures and images for: Exploring the Conserved Role of MANF in the Unfolded Protein Response in Drosophila melanogaster
Source: PLoS One. 2016 Mar 14;11(3):e0151550. doi: 10.1371/journal.pone.0151550 (PMC4790953; doi:10.1371/journal.pone.0151550)

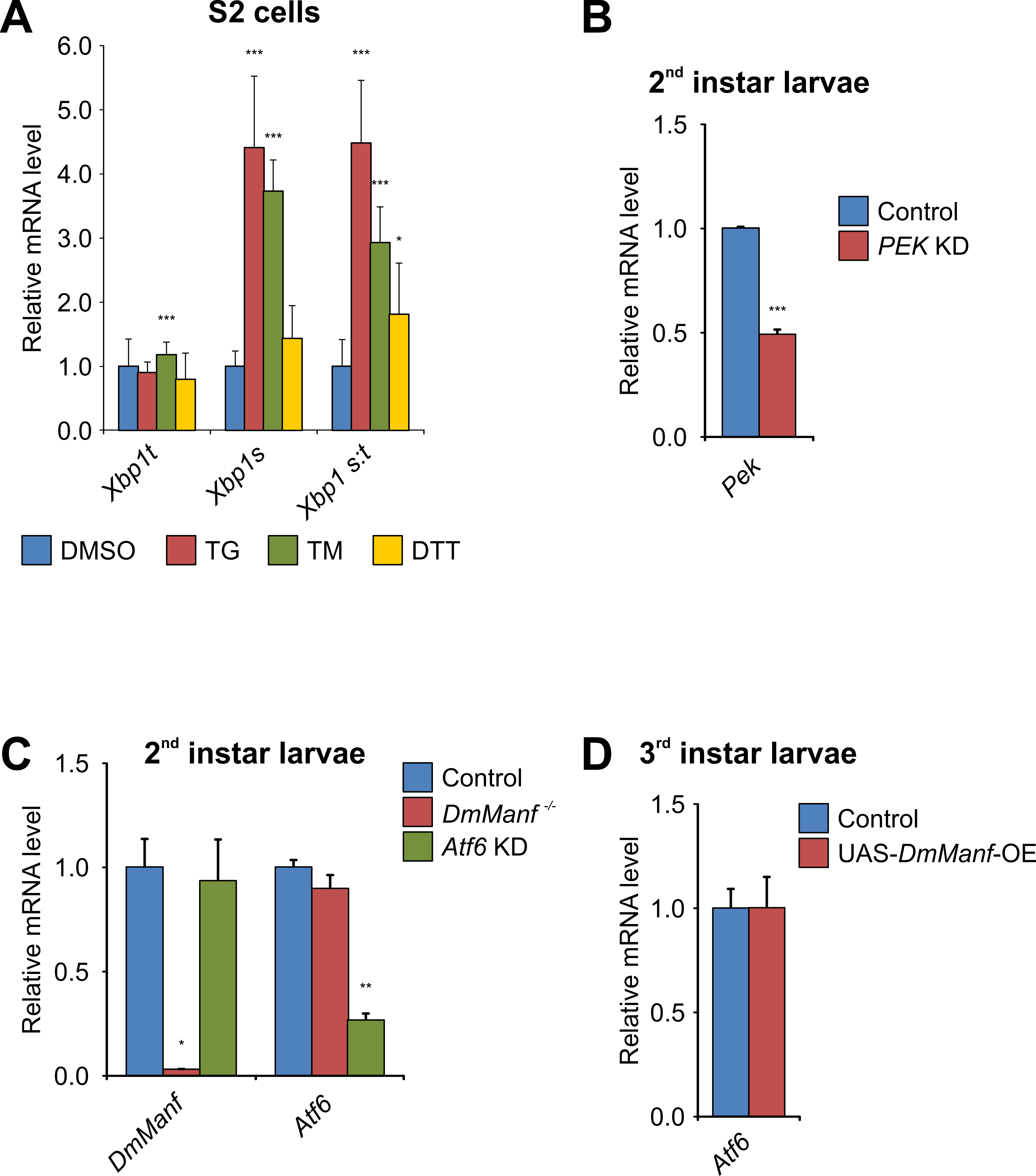

Supplement: S1 Fig — A) In Schneider 2 (S2) cells treated with ER stress-inducing drugs thapsigargin (TG), tunicamycin (TM) and dithiothreitol (DTT) the mRNA level of Xbp1s is increased while Xbp1t remains unaltered resulting in increase of Xbp1 s:t ratio. Xbp1t, total amount of Xbp1; Xbp1s, spliced-specific transcript of Xbp1; Xbp1 s:t, proportion of Xbp1s out of Xbp1t. B) In ubiquitous PEK knockdown larvae, mRNA level of PEK was decreased. C) The Atf6 mRNA level was not altered in zygotic DmManf mutants. Ubiquitous knockdown of Atf6 showed decreased expression level of Atf6 but did not alter DmManf mRNA expression. D) In 3rd instar wandering larvae, ubiquitous DmManf overexpression did not affect Atf6 expression. KD, knockdown. Average ± standard deviation. *, P<0.05; **, P<0.01; ***, P<0.001 versus control, Student’s t-test. (TIF) [file pone.0151550.s001.tif]

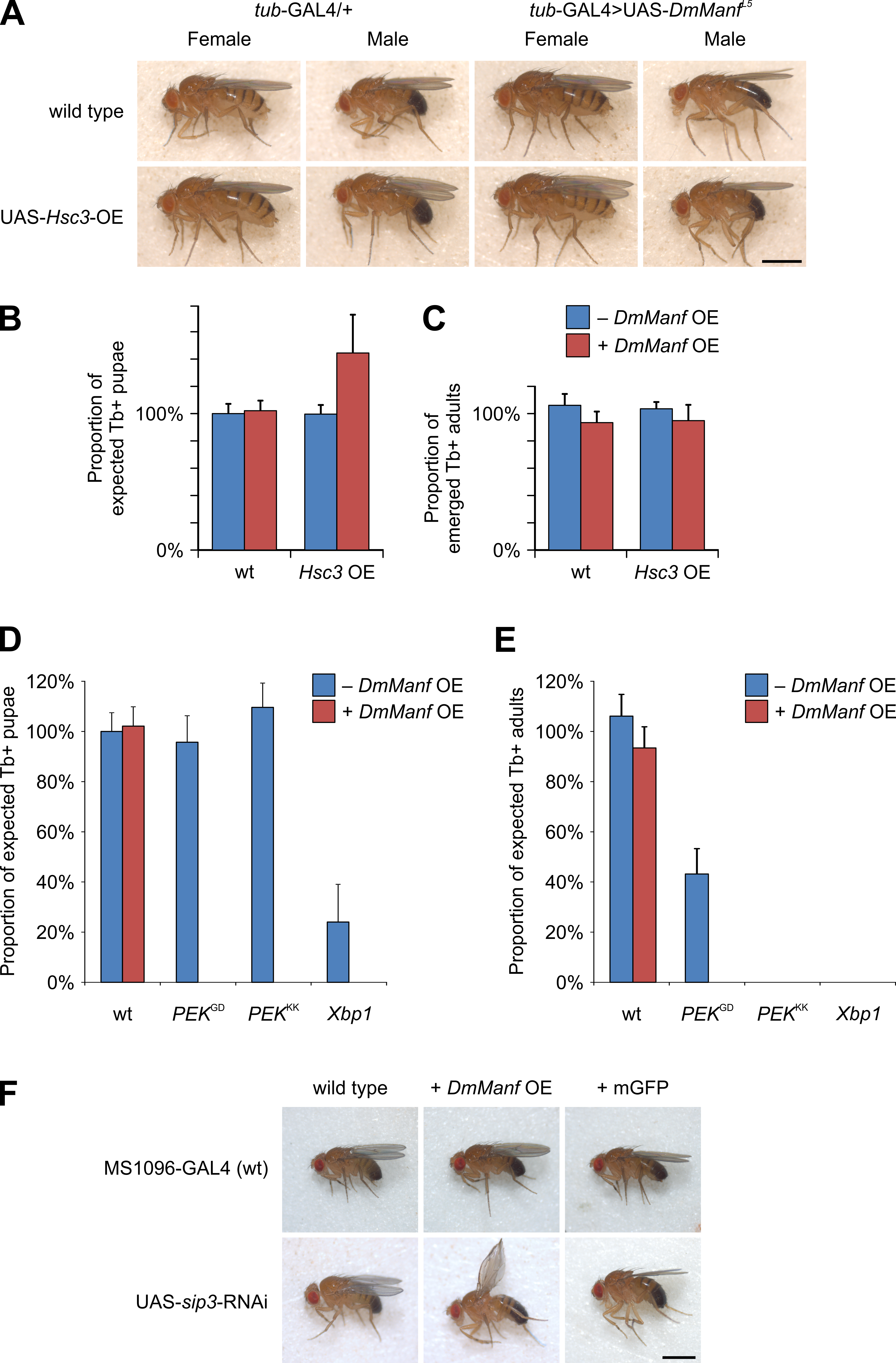

Supplement: S2 Fig — A–C) Ubiquitous overexpression of Hsc3 does not affect fly viability. A) Overexpression of DmManf or Hsc3 with tub-GAL4 showed no phenotype in adult flies. B–C) Viability of Hsc3 overexpression pupae (B) or adults (C) was not affected by overexpression of DmManf. D–E) Ubiquitous knockdown of PEK and Xbp1 with tub-GAL4 was viable (PEK) and only partially lethal (Xbp1) at pupal stage (D). Knockdown of PEK was also partially viable at adult stage (E). With DmManf overexpression, the ubiquitous knockdown of both PEK and Xbp1 was completely lethal at larval stage. F) Knockdown of sip3 with wing driver MS1096-GAL4 results in wrinkled wing phenotype in DmManf-overexpressing background. Scale bar 1 mm (in A and F). Amount of pupae analysed in B–C and D–E are presented in S3 Table. Proportion of Tb+ pupae was normalized to experimentally determined proportion of Tb+ pupae (see S3 Table, wild type and wild type/SM6-TM6). OE, overexpression. (TIF) [file pone.0151550.s002.tif]
