# Supplementary material for: Exploring the Conserved Role of MANF in the Unfolded Protein Response in Drosophila melanogaster
Source: PLoS One. 2016 Mar 14;11(3):e0151550. doi: 10.1371/journal.pone.0151550 (PMC4790953; doi:10.1371/journal.pone.0151550)
Supplement: S1 Table — Symbols used: Tf ID, transformant line identification; Collection, RNAi library where GD = Vienna Drosophila RNAi Center (VDRC) GD library, KK = VDRC KK library, BL = TRiP-3 collection available in Bloomington Drosophila Stock Center. According to the VDRC datasheet, the UAS-Xbp1-RNAi construct in transformant line 109312 targets both unspliced and spliced Xbp1 transcripts. (DOCX) [file pone.0151550.s003.docx]

**S1 Table. List of UAS-RNAi lines used in the study.**

| **Gene name** | **Symbol** | **CG number** | **Tf ID** | **Collection** |
| --- | --- | --- | --- | --- |
| *Heat shock protein cognate 3* | *Hsc3* | CG4147 | BL32402 | BL |
| *Heat shock protein cognate 3* | *Hsc3* | CG4147 | 14882 | GD |
| *Heat shock protein cognate 3* | *Hsc3* | CG4147 | 101766 | KK |
| *pancreatic eIF-2alpha kinase* | *PEK* | CG2087 | 16427 | GD |
| *pancreatic eIF-2alpha kinase* | *PEK* | CG2087 | 110278 | KK |
| *X box binding protein-1* | *Xbp1* | CG9415 | 109312 | KK |
| *septin interacting protein 3* | *sip3* | CG1937 | 107060 | KK |
| *Calcium ATPase at 60A* | *Ca-P60A* | CG3725 | 4474 | GD |
| *eukaryotic translation Initiation Factor 2alpha* | *eIF2-alpha* | CG9946 | 104562 | KK |
| *von Hippel-Lindau* | *Vhl* | CG13221 | 108920 | KK |
| *Inositol-requiring enzyme-1* | *Ire-1* | CG4583 | 39562 | GD |
| *Atf6* | *Atf6* | CG3136 | 36504 | GD |
| *Activating transcription factor 3* | *Atf3* | CG11405 | 26741 | GD |
| *TNF-receptor-associated factor 6* | *Traf6* | CG10961 | 110266 | KK |
| *cryptocephal* | *crc* | CG8669 | 25985 | GD |
| *Hrd3* | *Hrd3* | CG10221 | 1163 | GD |
| *Protein disulfide isomerase* | *Pdi* | CG6988 | 23358 | GD |

Symbols used: Tf ID, transformant line identification; Collection, RNAi library where GD = Vienna *Drosophila* RNAi Center (VDRC) GD library, KK = VDRC KK library, BL = TRiP‑3 collection available in Bloomington *Drosophila* Stock Center. According to the VDRC datasheet, the UAS-*Xbp1*-RNAi construct in transformant line 109312 targets both unspliced and spliced *Xbp1* transcripts.
