# Supplementary material for: Exploring the Conserved Role of MANF in the Unfolded Protein Response in Drosophila melanogaster
Source: PLoS One. 2016 Mar 14;11(3):e0151550. doi: 10.1371/journal.pone.0151550 (PMC4790953; doi:10.1371/journal.pone.0151550)
Supplement: S2 Table — Hsc3 and Pek primers were designed with FlyPrimerBank (http://www.flyrnai.org/FlyPrimerBank) [58]. DmManf and RpL32 were adopted from [20], Xbp1t from [59], Xbp1s from [60] and Atf6 from [61]. (DOCX) [file pone.0151550.s004.docx]

**S2 Table. List of primer pairs used in the qPCR analysis and their PCR efficiencies (E).**

| **Gene** | **Forward primer** | **Reverse primer** | **E** |
| --- | --- | --- | --- |
| *DmManf* | AATCTGCGACCTTCGCTATG | TCGTTGAGGATTTTCTTCAGG | 1.98 |
| *RpL32* | CGGATCGATATGCTAAGCTGT | GCGCTTGTTCGATCCGTA | 1.97 |
| *Hsc3* | GATTTGGGCACCACGTATTCC | GGAGTGATGCGGTTACCCTG | 1.94 |
| *Xbp1t* | TCTAACCTGGGAGGAGAAAG | GTCCAGCTTGTGGTTCTTG | 2.00 |
| *Xbp1s* | CCGAACTGAAGCAGCAACAGC | GTATACCCTGCGGCAGATCC | 2.00 |
| *Pek* | TACTAGGTCCAGTGGTGC | GCTTGTCCAGGTGGGAAGCTA | 1.60 |
| *Atf6* | AACGTAATTCCACGGAAGCCCAACA | GCGACGGTAGCTTGATTTCTAGAGCC | 1.82 |

*Hsc3 and Pek* primers were designed with FlyPrimerBank (http://www.flyrnai.org/FlyPrimerBank) (Hu *et al*. 2013). *DmManf* and *RpL32* were adopted from Palgi *et al*. 2012, *Xbp1t* from Benosman *et al*. 2013, *Xbp1s* from Maor *et al*. 2013 and *Atf6* from Kim *et al*. 2015.
