# Supplementary material for: Exploring the Conserved Role of MANF in the Unfolded Protein Response in Drosophila melanogaster
Source: PLoS One. 2016 Mar 14;11(3):e0151550. doi: 10.1371/journal.pone.0151550 (PMC4790953; doi:10.1371/journal.pone.0151550)
Supplement: S3 Table — tub-GAL4/TM6 Tb Sb EYFP females were crossed to UAS-x-RNAi (wild type background), UAS-x-RNAi; UAS-DmManf-OE/SM6-TM6 (+ UAS-DmManf-OE) or UAS-x-RNAi; UAS-mCD8-GFP/SM6-TM6 (+ UAS-mCD8-GFP) males. Since UAS-Hsc3-RNAiBL construct was inserted in 3rd chromosome and the insertion was lethal, UAS-Hsc3-RNAiBL/SM6-TM6 and UAS-DmManf-OE; UAS-Hsc3-RNAiBL/SM6-TM6 males were used. Columns: Tb+ and Tb-, amounts of Tb+ and Tb- pupae in crosses; Pupae, normalized proportion of Tb+ of all pupae, wild type or wild type/SM6-TM6 were used to normalize proportions; Adults, proportion of emerged adults out of Tb+ pupae. OE, overexpression; ND, not determined. n of analysed vials = 6 (except 1, n = 2 vials). (DOCX) [file pone.0151550.s005.docx]

**S3 Table. Results from ubiquitous knockdown studies of UAS-RNAi lines.**

|  | **wild type background** | | | | **+ UAS-*DmManf*-OE** | | | | **+ UAS-mCD8-GFP** | | | |
| --- | --- | --- | --- | --- | --- | --- | --- | --- | --- | --- | --- | --- |
| **UAS line** | **Tb+** | **Tb-** | **Pupae** | **Adults** | **Tb+** | **Tb-** | **Pupae** | **Adults** | **Tb+** | **Tb-** | **Pupae** | **Adults** |
| UAS-*Hsc3*-RNAi^GD 1^ | 0 | 126 | 0% | 0% | 0 | 113 | 0% | 0% | 0 | 178 | 0 | ND |
| UAS-*Hsc3*-RNAi^BL^ | 0 | 250 | 0% | 0% | 0 | 224 | 0% | 0% | ND | ND | ND | ND |
| UAS-*sip3*-RNAi ^1^ | 0 | 221 | 0% | 0% | 0 | 137 | 0% | 0% | 0 | 268 | 0 | ND |
| UAS-*Xbp1*-RNAi | 88 | 586 | 24% | 0% | 0 | 303 | 0% | 0% | 35 | 421 | 25% | ND |
| UAS-*PEK*-RNAi^GD^ | 456 | 443 | 96% | 43% | 0 | 332 | 0% | 0% | 184 | 350 | 108% | ND |
| UAS-*PEK*-RNAi^KK^ | 590 | 423 | 110% | 0% | 0 | 356 | 0% | 0% | 227 | 332 | 128% | ND |
| UAS-*Hsc3*-OE | 409 | 369 | 100% | 104% | 331 | 384 | 144% | 95% | ND | ND | ND | ND |
| wild type | 489 | 423 | 100% | 106% | 444 | 359 | 102% | 93% | 443 | 394 | 99% | ND |
| wild type/SM6-TM6 | 138 | 279 | 100% | 106% | ND | ND | ND | ND | ND | ND | ND | ND |

*tub*-GAL4/TM6 Tb Sb EYFP females were crossed to UAS-*x*-RNAi (wild type background), UAS-*x*-RNAi ; UAS-*DmManf*-OE/SM6-TM6 (+ UAS-*DmManf*-OE) or UAS-*x*-RNAi ; UAS-mCD8-GFP/SM6-TM6 (+ UAS-mCD8-GFP) males. Since UAS-*Hsc3*-RNAi^BL^ construct was inserted in 3^rd^ chromosome and the insertion was lethal, UAS-*Hsc3*-RNAi^BL^/SM6-TM6 and UAS-*DmManf*-OE ; UAS-*Hsc3*-RNAi^BL^/SM6-TM6 males were used. Columns: Tb+ and Tb-, amounts of Tb+ and Tb- pupae in crosses; Pupae, normalized proportion of Tb+ of all pupae, wild type or wild type/SM6-TM6 were used to normalize proportions; Adults, proportion of emerged adults out of Tb+ pupae. OE, overexpression; ND, not determined. n of analysed vials = 6 (except ^1^, n = 2 vials).
